# Comparison of genome-wide association and genomic prediction methods for milk production traits in Korean Holstein cattle

**DOI:** 10.5713/ajas.18.0847

**Published:** 2019-02-09

**Authors:** SeokHyun Lee, ChangGwon Dang, YunHo Choy, ChangHee Do, Kwanghyun Cho, Jongjoo Kim, Yousam Kim, Jungjae Lee

**Affiliations:** 1 Animal Breeding and Genetics Division, National Institute of Animal Science, RDA, Cheonan 31000, Korea; 2 Division of Animal and Dairy Science, Chungnam National University, Daejeon 34134, Korea; 3 Department of Dairy Science, Korea National College of Agriculture and Fisheries, Jeonju 54874, Korea; 4 Division of Applied Life Science, Yeungnam University, Gyeongsan 38541, Korea; 5 Jun P&C Institute, INC., Yongin 16950, Korea

**Keywords:** Bayesian Approach, Genomic Selection, Holstein Cattle, Milk Production, Single-step Genomic Best Linear Unbiased Prediction

## Abstract

**Objective:**

The objectives of this study were to compare identified informative regions through two genome-wide association study (GWAS) approaches and determine the accuracy and bias of the direct genomic value (DGV) for milk production traits in Korean Holstein cattle, using two genomic prediction approaches: single-step genomic best linear unbiased prediction (ss-GBLUP) and Bayesian Bayes-B.

**Methods:**

Records on production traits such as adjusted 305-day milk (MY305), fat (FY305), and protein (PY305) yields were collected from 265,271 first parity cows. After quality control, 50,765 single-nucleotide polymorphic genotypes were available for analysis. In GWAS for ss-GBLUP (ssGWAS) and Bayes-B (BayesGWAS), the proportion of genetic variance for each 1-Mb genomic window was calculated and used to identify informative genomic regions. Accuracy of the DGV was estimated by a five-fold cross-validation with random clustering. As a measure of accuracy for DGV, we also assessed the correlation between DGV and deregressed-estimated breeding value (DEBV). The bias of DGV for each method was obtained by determining regression coefficients.

**Results:**

A total of nine and five significant windows (1 Mb) were identified for MY305 using ssGWAS and BayesGWAS, respectively. Using ssGWAS and BayesGWAS, we also detected multiple significant regions for FY305 (12 and 7) and PY305 (14 and 2), respectively. Both single-step DGV and Bayes DGV also showed somewhat moderate accuracy ranges for MY305 (0.32 to 0.34), FY305 (0.37 to 0.39), and PY305 (0.35 to 0.36) traits, respectively. The mean biases of DGVs determined using the single-step and Bayesian methods were 1.50±0.21 and 1.18±0.26 for MY305, 1.75±0.33 and 1.14±0.20 for FY305, and 1.59±0.20 and 1.14±0.15 for PY305, respectively.

**Conclusion:**

From the bias perspective, we believe that genomic selection based on the application of Bayesian approaches would be more suitable than application of ss-GBLUP in Korean Holstein populations.

## INTRODUCTION

High production ability has been used for primary selection in dairy breeding schemes. In particular, milk yield, fat yield, and protein yield are the most important economic traits for dairy cattle selection. To date, genetic improvement of these economic traits has been performed successfully based on traditional best linear unbiased prediction (BLUP), and the breeding values of economic traits have been applied with selection indices in Korean dairy breeding systems. The BLUP used in combination with individual records and estimated breeding value (EBV) has resulted in considerable genetic progress in the dairy industry [1]. In recent years, however, genomic information in the form of commercial single-nucleotide polymorphic (SNP) marker panels from various companies (i.e., Illumina, San Diego, CA, USA; Neogen-GeneSeek, Lincoln, NE, USA; and Affymetrix, Santa Clara, CA, USA) have become available for genetic evaluations, as a consequence of improvements in genotyping technology and statistical methods after introduction by Meuwissen et al [2] in 2001. Accordingly, genomic prediction using genotypic data has been widely applied for various livestock.

Genomic selection (GS) involves selection of bulls based on genomic breeding values, which are derived from the combination of EBVs and direct breeding values (DGVs) based on SNPs using several blending formulae [3,4] or single-step methods (e.g., single-step genomic best linear unbiased prediction [ss-GBLUP]) [5] and single-step super hybrid model [6]). The advantages of GS are simplicity and resistance to preselection bias [7,8] and more reliable prediction than traditional BLUP [1,9,10]. When GS schemes are applied in the field, the use of young bulls should be the most effective in terms of reliability. For example, in young Holstein bulls in the United States, reliabilities for predicted transmitting abilities for milk yield based on genomic information ranged from 73% to 79% [11].

Typically, there are two approaches to performing GS. The first method is multiple-step GS. In step 1 of this method, pseudo-phenotypes (i.e., EBV or deregressed-EBVs), which include information related to genotyped and ungenotyped animals, are calculated for the genotyped animals; in step 2, DGV is calculated using the pseudo-records and genotyped data (i.e., Bayesian and GBLUP approaches); and in step 3, the traditional EBV and DGV are combined into genomic-enhanced EBVs (GE-EBVs). The second method is ss-GBLUP. To construct a blended relationship matrix (H-matrix) [5] using ss-GBLUP, a numeric relationship matrix (NRM) is replaced with a genomic relationship matrix (GRM) and then these can be blended with an NRM [10]. In ss-GBLUP, the accuracy obtained for milk yield is greater than that obtained using multiple-step GS [10]. However, a drawback of ss-GBLUP is that it cannot be applied to non-linear estimates, although some solutions to ss-GBLUP non-linear estimations have been presented in the literature [10].

The objectives of this study were to compare identified informative regions through two different genome-wide association study (GWAS) approaches and assess the accuracy and bias of DGVs for milk production in Korean dairy cattle using genomic prediction approaches (i.e., ss-GBLUP and Bayesian).

## MATERIALS AND METHODS

### Phenotypic data

Raw data for the period from 1998 to 2018 were obtained from data collected by the National Agricultural Cooperative Federation’s dairy cattle improvement center by way of its milk testing program, which is nationally based. The pedigree data for this analysis were obtained from the Korean Animal Improvement Association. Traits considered in this study were adjusted 305-day (d) milk yield (MY305), adjusted 305-d fat yield (FY305), and adjusted 305-d protein yield (PY305). The data set included records for Holstein cows in the first parity with full pedigree information and excluded records with extreme milk production (MY305, <2,500 or >16,000 kg; FY305, <70 or >600 kg; and PY305, <80 or >500 kg), age at calving (<17 or >31 months). The final number of edited records was 265,271. Table 1 shows the basic statistics of the data.

### Genotypic data

Genotypic data were obtained using two SNP panels: BovineSNP50 v2 and BovineSNP50 v3 (Illumina Inc., USA). These two SNP panels were imputed to BovineSNP50 version 3 using Fimpute version 2.2 [12]. After excluding unmapped SNPs and SNPs on sex chromosomes, the available number of SNP markers was 54,931. After performing marker quality control, genotypes at each locus were excluded based on the following criteria: average call rate lower than 0.90; minor allele frequency less than 0.01; markers not in Hardy–Weinberg equilibrium, with a chi-square value (χ^2^) greater than 95%; and SNPs in extreme linkage disequilibrium (LD, r^2^ >0.99). After editing, 50,765 SNP genotypes were available for analysis. Furthermore, genotyped animals were excluded from analysis based on the following criteria: duplicate animals, twin animals, and animals that failed parentage tests. Duplicate animals and twin animals were removed based on marker call rates. Furthermore, genotype identification that could not be matched to the corresponding animal in the phenotypic data set was removed from a total of 2,032 Holstein dairy cattle. Finally, for ss-GBLUP for all traits, the genotype data set comprised 1,919 animals, whereas for Bayes-B, the number of animals available for MY305, FY305, and PY305 was 963, 943, and 946, respectively.

### Statistical model

Genetic components, breeding values, and corresponding reliabilities of milk production traits were estimated using following mixed-model equation:

$$y_{ijk}={HYS}_{i}+{age}_{j}+a_{k}+e_{ijk}$$

where y_ijk_ is the observation; HYS_i_ is the fixed effect of the ith herd-year season; age_j_ is the fixed effect of the jth calving age; a_k_ is the random genetic effect of animal k; and e_ijk_ is the residual effect. Using a univariate animal model, the covariance between traits was assumed to be zero. In matrix notation, the statistical model with single traits was as follows:

y=Xb+Za+e

where **y** is the matrix of observations for the traits; **X** and **Z** are the known incidence matrices for fixed and random effects; ***b*** is the vector of fixed effects; ***a*** is the vector of additive genetic effects for each animal, and ***e*** is the vector of the residual effect.

Total phenotypic variance ($\sigma_{p}^{2}$) was defined as the sum of additive ($\sigma_{a}^{2}$), and residual ($\sigma_{e}^{2}$) variance. Thus, the heritability was calculated as $h^{2}=\frac{\sigma_{a}^{2}}{\sigma_{p}^{2}}$, where *h*^2^ is the estimate of heritability.

The reliability of breeding value was then calculated as: $\sqrt{1-PEV/\sigma_{a}^{2}}$, where PEV is the prediction error variance. The variance components and EBVs were estimated using the expectation maximization restricted maximum likelihood (EM-REML) algorithm in the REMLF90 and the BLUPF90 software module from the BLUPF90 family [13].

#### Single-step method

The ss-GBLUP was used to predict DGVs (DGV_ss_) and analyze genome-wide association study data (ssGWAS). The ss-GBLUP method is a modification of BLUP. The numerator relationship matrix (A^−1^) was replaced by an **H^−1^** matrix (a combination of numerator relationship matrix and GRM) as follows:

$$H^{-1}=A^{-1}+\left[\begin{array}{cc} 0 & 0\\ 0 & G^{-1}-A_{22}^{-1} \end{array}\right]$$

where A_22_ is a numerator relationship matrix for genotyped animals and G is a GRM. The genomic matrix (G) is formed based on [3] as follows:

$$\text{G}=\text{ZD}{\text{Z}}^{\prime }\text{q}$$

where Z is the incidence matrix for markers, D is a weight matrix for SNPs (initially D = I), and q is a weighting factor. The weighting factor can be obtained by using either SNP frequency [3] or by guaranteeing that the average diagonal in G approaches that of A_22_ [8]. In the present study, for increasing the weights of SNPs with large effects and decreasing those with small effects, the SNP effect and weighting factor were derived using several steps, which are described by Wang et al [14]. In the present study, the weighting factor used second iteration and all procedures were performed using the BLUPF90 family [13].

#### Bayesian method

Deregressed-estimated breeding value (DEBV) adjusting for parental average (DEBV-PA) values, which contained only phenotypic information for individuals and their descendants used for deregression (dividing by the reliability of EBV) with parental information [9], were used as response variables for prediction of DGV_Bayes_ and GWAS (BayesGWAS) in the multiple-step process. To ensure the quality of DEBV-PA values, those animals with a reliability of less than 0.10 were removed. To account for the heterogeneous variance of DEBV, the response variable was weighted because each animal has different reliabilities. The weighting factor (*w_i_*) [15] for animal *i* was calculated as follows:

$$w_{i}=\frac{\left(1-h^{2}\right)}{\{c+[(1-r_{i}^{2})/r_{i}^{2}]\}h^{2}}$$

where $r_{i}^{2}$ is the reliability of EBV, *h*^2^ is the heritability of the trait, and *c* is the the proportion of genetic variation that could not be explained by the genetic information (i.e., SNP markers). In this study, *c* was assumed to be equal to 0.40 [16].

To estimate SNP marker effects, the Bayes-B method was used [2] with π set to 0.99. The Bayes-B method assumes that some proportion (π) of SNP markers has zero effects and that each SNP marker has locus-specific variance, which contrasts with the Bayes-C method. For each trait, marker effects were estimated using the following model equation:

$$y_{i}=\mu +\sum_{j=1}^{k}Z_{ij}u_{j}\delta_{j}+e_{i}$$

where *y_i_* is DEBV on animal *i* for the respective trait; *μ* is the population mean; *k* is the number of markers; *Z_ij_* is the allelic state at locus *j* in individual *i*; *u_j_* is the random substitution effect for marker *j*, which follows a mixed distribution for this random substitution effect according to indicator variable (*δ_j_*), a random 0/1 variable indicating the absence or presence of marker *j* in the model, with *u_j_* assumed to be normally distributed $\text{N}(0,\sigma_{u}^{2})$ when *δ_j_* = 1; and *e_i_* is a random residual effect assumed to be normally distributed $\text{N}(0,\sigma_{e}^{2})$.

The posterior distributions of the parameters and effects were obtained using Gibbs sampling. We performed a Markov chain Monte Carlo (MCMC) simulation of 110,000 rounds with Gibbs sampling, of which the first 10,000 iterations were discarded as burn-in. To estimate posterior means and variances of marker effects, Metropolis–Hastings samples were run for 10 iterations. The prior genotypic and residual variances from the results of REML were used, which were implemented in the BLUPf90 family. All procedures were implemented using GenSel4R software [17].

### Genome-wide association study analysis

Detection of informative regions or loci based on single SNPs may result in noise or underestimation due to the high ratio between the number of SNPs and the number of genotyped animals [14], and adjacent SNPs may be in high LD with the same quantitative trait locus (QTL) in high-density SNP panels because the effect of the QTL would be spread over all SNPs in high LD [18]. For this reason, non-overlapping 1-Mb windows, which is the proportion of genetic variance in each region consisting of a 1-Mb genome window, were calculated and used to identify informative genomic regions accounting for LD, which is more appropriate than using single SNPs.

The significance level of the informative 1-Mb window re gion in ssGWAS and BayesGWAS was, respectively, 1.0% and 0.5% of additive genetic variance, which was estimated as a portion of the total genetic variance explained by all SNPs.

### Accuracy of the direct genomic value

To estimate the accuracy of DGVs, we applied five-fold cross-validation with random clustering, whereby we set up training data sets, each of which was each constructed by masking the phenotype in the SS-method (i.e., setting the phenotype of genotyped cows and daughters of genotyped sires and their “unknown”) and the response variable in the Bayesian method (i.e., setting the response variable “unknown”), whereby 20% of the total individuals is set to random without replacement so as to be masked precisely once in the training data sets. Using these steps, we produced five training and testing sets. This results in each genotyped animal having DGV_ss_ and DGV_Bayes_ values from the masking data set, as derived using the single-step and Bayesian methods, respectively. The correlation coefficient between the DGV and DEBV values was calculated and used as a measure of the accuracy of DGV. Additionally, the bias (spread) of DGV for each method was assessed using regression coefficients. Table 2 summarizes the number of masked animals and phenotypes in each data set.

## RESULTS AND DISCUSSION

### Genetic parameter estimation

Variance components and heritability were estimated from regular phenotypic BLUP based on a univariate animal model. The estimated heritabilities for MY305, FY305, and PY305 were 0.26, 0.21, and 0.22, respectively (Table 3).

Previous studies have obtained similar heritability estimates for MY305, FY305, and PY305 of 0.30, 0.28, and 0.25 [19] and 0.23, 0.19, and 0.19 [20], respectively.

### Genome-wide association study

**Figure 1:**
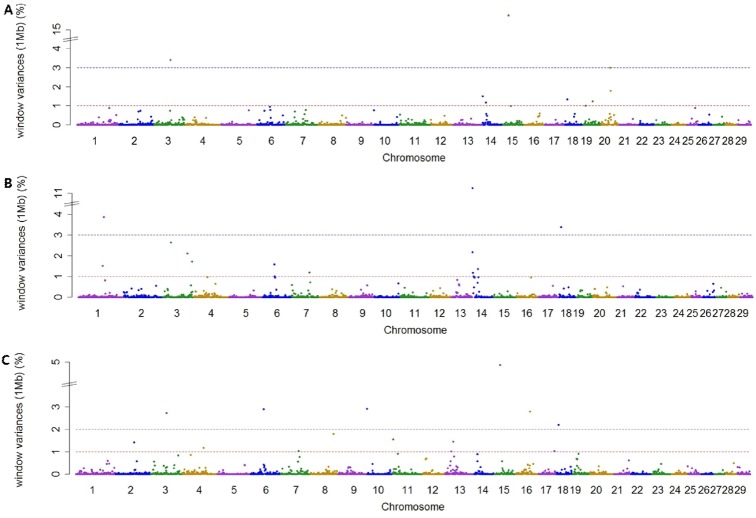
Manhattan plots showing genome-wide significant informative windows (≥1% threshold) for adjusted 305-day milk yield (A), adjusted 305-day fat yield (B), and adjusted 305-day protein yield (C) in Korean Holstein cattle using the single-step method.

**Figure 2:**
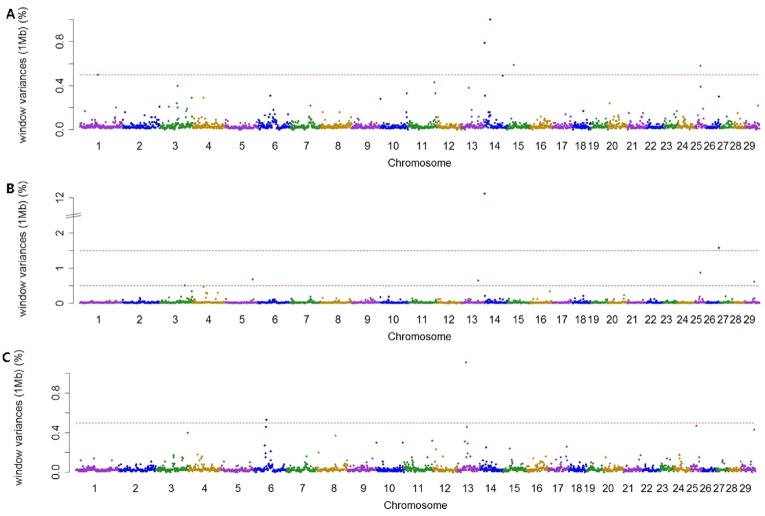
Manhattan plots showing genome-wide significant informative windows (≥0.5% threshold) for adjusted 305-day milk yield (A), adjusted 305-day fat yield (B), and adjusted 305-day protein yield (C) in Korean Holstein cattle using the BayesB method.

Using association analysis based on ssGWAS and BayesGWAS, we detected the most significant regions for SNP markers on the Illumina BovineSNP50 panel. Figures 1, 2 shows plots of genetic variance accounted for by 1-Mb windows, within a chromosome, based on different methods. Table 4 shows the results of GWAS for milk production traits. The GWAS results include the chromosomal position and fraction of variance of 1-Mb genome windows by informative regions (greater than 0.5% or 1.0%). Using BayesGWAS, there were 2,521 regions, with an average number of 20 SNPs, whereas for ssGWAS, there were 2,024 regions with an average number of 20 SNPs.

A total of nine and five significant windows (1-Mb) were identified for MY305 using ssGWAS and BayesGWAS, respectively. The most informative window was detected on chromosome *Bos taurus* autosomes 15 (BTA15) at 23 Mb using ssGWAS and on BTA14 at 21 Mb using BayesGWAS, which explained 15.73% and 1.0%, respectively. An informative window common to both ssGWAS and BayesGWAS was identified on BTA14 at 1 Mb, which explained 1.54% and 0.79%, respectively. For FY305, we detected 12 significant QTLs using ssGWAS and seven significant QTLs using BayesGWAS. The region of BTA14 at 1 Mb was the most significant 1-Mb window region and a common significant region detected using both methods, which indicated that 11.25% (ssGWAS) and 12.12% (BayesGWAS) of the additive genetic variance was captured, respectively. For PY305, we identified 14 and two significant regions using ssGWAS and BayesGWAS, respectively. Using ssGWASs and BayesGWAS, the most informative window was detected on BTA15 at 24 Mb and on BTA13 at 31 Mb, respectively. A common informative window obtained using both methods was detected on BAT13 at 31 Mb.

The BTA14 region has received considerable attention from many scientists as this region has been reported to harbor a large number of QTLs having an effect on milk production. The diacylglycerol O-acyltransferase 1 (*DGAT1*) gene located at 1 Mb on BTA14 is generally accepted to be a major gene for milk production [21,22]. In addition to the *DGAT1* gene, the 1-Mb region of BTA14 also harbors a number of other genes with linkage to *DGAT1*, such as cytochrome P450 family 11 subfamily B member 1 [22,23]. Accordingly, using both ssGWAS_s_ and BayesGWAS, the 1 Mb region of BTA14 was identified to be a region associated milk and fat yield. Although the 1-Mb region on BTA14 has also previously been shown to be informative with respect to milk protein [21,22], in the present study, we were unable to detect this window with regards to milk protein. This could be attributable to the fact that the collection system for milk protein yield data in Korea was recently changed due to problems associated with the standard solution used. Accordingly, the data for milk protein are not standardized. Therefore, further research is required to obtain uniform milk protein data.

Our findings relating to the 1-Mb region on BTA14, along with other significant regions, are consistent with previously identified regions that have a potential influence on milk production in the Animal QTL database (https://www.animalgenome.org/cgi-bin/QTLdb/BT/index).

Despite the significantly higher level of genetic variance associated with using ssGWAS than when using BayesGWAS, the former was able to identify a larger number of significant regions. Moreover, it is notable that few significant regions were detected using both ssGWAS and BayesGWAS approaches, which can probably be attributed to the differences in methodologies. Methods like Bayes-B are strongly affected by priors, and by the proportion of SNPs assumed not to have an effect (π) [14,15,24]. In contrast to Bayesian methods, ssGWAS analysis is based on available pedigree relationships, and does not depend on deregression [14]. Previous studies have investigated different GWAS approaches using simulated data sets, and found that the different methods were able to detect the same regions [25,26]. In contrast, however, Wang et al [14] found that few common regions were detected using different methods. These disparate findings can probably be explained in terms of the limitations of simulations, which do not capture the complexities of real data.

### Accuracy of direct genomic value

On the basis of our previous GWAS results, we identified common QTL regions using two different approaches (i.e., ss-GBLUP and Bayes-B). However, we were unable to accurately determine the location and effect size of true QTLs. Therefore, we also compared the accuracy and bias of DGVs when using the two approaches.

Table 5 shows the accuracy and bias of the DGVs deter mined using the ss-GBLUP (single-step method) and Bayes-B (Bayesian method) approaches. To gain estimates of the accuracy and degree of bias of DGVs, we calculated the averages of correlation and regression coefficients in predicting the masking individual in the validation set for analysis of the non-masking individual in the training set, respectively.

The mean accuracies of DGV _ss_ and DGV_Bayes_ for MY305, FY305, and PY305 were 0.316±0.018, 0.374±0.070, and 0.354± 0.051, and 0.335±0.034, 0.389±0.052, and 0.357±0.033, respectively. The mean biases of DGVs detected using the single-step method were 1.497±0.210 (MY305), 1.745±0.3266 (FY305), and 1.585±0.203 (PY305), whereas those using the Bayesian method were 1.182±0.262 (MY305), 1.138±0.199 (FY305), and 1.135±0.145 (PY305).

For the three studied traits, we noted small differences in the accuracy of the DGVs obtained using the two methods. The prediction accuracy for trait MY305 (Milk [Acc.]) was lower than that for the other milk production traits (fat and protein). However, compared with Bayes-B, the single-step method for MY305, FY305, and PY305 had a higher bias. By using weighting factors in the Bayes-B method, the more reliably genotyped animals made a greater contribution in estimating SNP marker effects and the prediction of DGVs. We did not apply weighting factors when using ss-GBLUP as real phenotypes were used as response variables when using this method.

A direct comparison of the accuracy and bias of DGV de termined in the present study with those determined previously is difficult given differences in populations and methodologies, such as clustering methodologies (e.g., K-means vs random vs identity by state IBS clustering), the models used, assessments of method accuracy (e.g., genetic correlation vs simple vs variable setting), and other reasons [9]. Furthermore, accuracy depends on various parameters, including the reference population size and its genetic structure [27]. In this regard, in a previous study on Danish Holsteins using a five-fold cross-validation, Su et al [28] reported that the accuracy of DGV (r_DGV,EBV_) for milk production ranged from 0.64 to 0.70. Similarly, Ding et al [29] in their study of Chinese Holsteins, reported that the accuracy of DGV (r_DGV, EBV_) in five-fold cross-validation using Bayes-B with priors (π = 0.99) and GBLUP for milk production ranged from 0.317 to 0.380, whereas Luan et al [30] reported an accuracy for milk production of 0.54 to 0.56 in their study on Norwegian red cattle.

We found that the mean accuracies of DGVs for milk pro ductions in the present study were smaller than those obtained previously, which can probably be explained by the fact that the reference population size in our study was smaller than that used in other studies, which was at least 2,000 bulls. Therefore, we intend to increase the size of our reference population by continuously updating data on genotyped animals and phenotypes. This will accordingly improve the accuracy of our genomic predictions. Similarly, if real variants (true QTLs) identified from putative informative regions based on GWAS results can be sequenced in detail, this will enhance the accuracy of genomic prediction.

## CONCLUSION

In this study, we compared the informative regions identified by GWAS and the accuracy of DGV between multiple approaches. We found that different numbers of informative regions were detected when using single-step and Bayesian approaches, and that few common regions were identified by both methods. However, a 1-Mb region on chromosome BTA14, which is known to harbor many genes, was identified by both methods. The mean accuracy of DGVs for milk production traits was similar for both methods, although Bayes-B tended to show a relatively lower bias than the ss-GBLUP method. Therefore, from the perspective of bias, we believe that a Bayesian approach (i.e., Bayes-B) would be more suitable in GS for Korean Holstein populations.

## Figures and Tables

**Table 1:** Basic statistics of milk composition

Traits	N	Mean	SD	Min	Max
MY305	265,271	8,437.50	1,718.70	2,504	15,962
FY305	265,004	321.28	73.11	70	600
PY305	261,021	269.02	52.94	80	500

SD, standard deviation; MY305, adjusted 305-d milk yield; FY305, adjusted 305-d fat yield; PY305, adjusted 305-d protein yield.

**Table 2:** Number of masking animals and phenotypes

Item	Single-step GBLUP			Number of masking genotyped animal	Bayesian approach		
	
	Number of masking phenotype				Number of masking genotyped animal		
	
	MY305	FY305	PY305		MY305	FY305	PY305
1	16,229	16,197	16,230	398	196	192	194
2	12,438	12,401	12,441	391	191	186	188
3	11,940	11,892	11,937	386	193	189	191
4	14,444	14,418	14,442	362	188	183	184
5	7,792	7,778	7,791	382	196	193	193
Total	265,271	265,004	261,021	1,919	963	943	946

GBLUP, genomic best linear unbiased prediction; MY305, adjusted 305 d milk yield; FY305, adjusted 305 d fat yield; PY305, adjusted 305 d protein yield.

**Table 3:** Variance components, standard error, and heritability estimates for milk production in Korean Holstein cattle

Trait	Additive genetic variance	Residual variance	Heritability
MY305	416,220 (±12,855)	1,204,200 (±10,621)	0.26
FY305	514.23 (±18.79)	1,947.8 (±15.89)	0.21
PY305	307.44 (±11.20)	1,102.4 (±9.40)	0.22

MY305, adjusted 305-d milk yield; FY305, adjusted 305-d fat yield; PY305, adjusted 305-d protein yield.

**Table 4:** Result of GWAS for milk production traits

Method	Trait	Chr_Mb	gV (%)	Total SNP	Method	Trait	Chr_Mb	gV (%)	Total SNP
	
Single-step	MY305	15_23	15.73	61	Bayes B	MY305	14_21	1.00	18
		3_65	3.41	27			14_1	0.79	15
		20_37	3.01	24			15_24	0.59	60
		20_38	1.80	29			25_20	0.58	18
		14_1	1.50	27			1_65	0.50	23
		18_7	1.34	27		FY305	14_1	12.12	15
		19_35	1.24	24			26_46	1.58	21
		14_15	1.18	25			25_20	0.87	18
		19_8	1.01	21			5_101	0.68	16
	FY305	14_1	11.25	28			13_61	0.65	16
		1_103	3.86	22			29_32	0.62	22
		18_7	3.38	29			3_92	0.52	28
		3_32	2.64	97		PY305	13_31	1.11	21
		14_2	2.17	23			6_45	0.53	22
		3_99	2.1	23					
		3_118	1.71	19					
		6_53	1.58	54					
		1_98	1.51	26					
		14_23	1.35	21					
		7_73	1.19	52					
		14_3	1.17	35					
	PY305	15_24	5.85	60					
		10_0	2.90	229					
		6_53	2.90	53					
		16_59	2.79	25					
		18_7	2.20	27					
		8_96	1.80	29					
		11_2	1.55	26					
		13_31	1.44	28					
		2_75	1.41	20					
		4_85	1.17	19					
		17_66	1.03	29					
		7_73	1.03	28					
		13_23	1.01	56					
		3_65	2.71	16					

GWAS, genome-wide association study; SNP, single-nucleotide polymorphic; MY305, adjusted 305-d milk yield; FY305, adjusted 305-d fat yield; PY305, adjusted-305protein yield.

**Table 5:** Accuracy and bias of DGV in the 5-fold cross-validation using single-step GBLUP and Bayes approach

Traits	Data set	Accuracy (r_DGV,DEBV_)		Bias (b_DEBV,DGV_)	
	
		single-step GBLUP	Bayes approach	single-step GBLUP	Bayes approach
MY305	Training 1	0.326	0.296	1.707	1.056
	Training 2	0.303	0.349	1.230	1.061
	Training 3	0.332	0.345	1.613	1.208
	Training 4	0.291	0.304	1.316	0.963
	Training 5	0.330	0.380	1.62	1.624
	Average (SD)	0.316 (±0.018)	0.335 (±0.034)	1.497 (±0.210)	1.182 (±0.262)
FY305	Training 1	0.324	0.358	1.618	1.031
	Training 2	0.466	0.462	1.808	1.239
	Training 3	0.29	0.328	1.296	0.856
	Training 4	0.377	0.381	1.812	1.197
	Training 5	0.414	0.417	2.191	1.368
	Average (SD)	0.374 (±0.070)	0.389 (±0.052)	1.745 (±0.3266)	1.138 (±0.199)
PY305	Training 1	0.394	0.363	1.852	1.113
	Training 2	0.403	0.399	1.743	1.115
	Training 3	0.377	0.360	1.507	1.041
	Training 4	0.298	0.305	1.383	1.022
	Training 5	0.298	0.36	1.440	1.384
	Average (SD)	0.354 (±0.051)	0.357 (±0.033)	1.585 (±0.203)	1.135 (±0.145)

DGV, direct genomic value; GBLUP, genomic best linear unbiased prediction; MY305, adjusted 305-d milk yield; SD, standard deviation; FY305, adjusted 305-d fat yield; PY305, adjusted 305-d protein yield.
